# Surgical duration as a factor in postoperative infection rates in orthopedic procedures

**DOI:** 10.6026/973206300211620

**Published:** 2025-06-30

**Authors:** Rakshana Munusamy, J. Naveen Bose, Anthony Peter Joseph, Kamalkant Arvindkumar Singh, Ajeet Saoji, Manju Priya Sivasamy, Goutham Murugesan

**Affiliations:** 1 Department of Surgery, Madurai Medical College, Madurai, Tamilnadu, India; 2 Department of Emergency Medicine, Madurai Medical College, Madurai, Tamilnadu, India; 3 Department of Multi-Speciality, Fortis Nagarbhavi Bangalore, Karnataka, India; 4 Department of Pediatrics, GMERS Medical College, Valsad, Gujarat, India; 5 Department of Community Medicine, N. K. P. Salve Institute Of Medical Sciences & Research Centre and Lata Mangeshkar Hospital, Nagpur, Maharashtra, India; 6 Department of Emergency Medicine, Sathiya Multispeciality Hospital, Chennai, Tamil Nadu, India; 7 Department of Intensive Care, Orthomed Hospital, Royapettah, Chennai, Tamilnadu, India

**Keywords:** Surgical duration, postoperative infections, orthopedic procedures, infection risk, patient outcomes

## Abstract

Postoperative infection rates have been reported to be extremely sensitive to the surgical duration, particularly for orthopedic procedures that often require more extensive time and therefore increased exposure to infectious agents. Therefore, it is of interest to assess the surgical time and infection rate postoperative for 100 patients who had different orthopedic procedures performed. Surgical duration, infection rate and predisposing factors of infection were explored to determine limits where infection is likely to rise. Results suggest that long surgical time is highly significantly related to an increased rate of postoperative infection therefore, efforts to make the operative time optimal are relevant. These findings have enlightened the necessity for optimizing surgical time to enhance orthopedic patient outcomes.

## Background:

Postoperative infections contribute to a wide range of increased periods of staying in the hospitals, costs and worsening outcomes post-orthopedic procedures as a major concern [1]. Many factors count in infection risks and one factor known to be as a modifiable risk factor for infection is surgical time [2]. Prolonged surgical times might expose the patient to increased levels of environmental contamination, deterioration in tissue integrity and weakening immunity due to prolonged anesthesia time and trauma that may result from surgical stress [3]. The use of implants and hardware is especially risky in orthopedic procedures and would increase the infection risk further [4]. The existing literature shows a positive correlation between prolonged surgical time and postoperative infection rates however, the threshold duration beyond which infection risk increases is inconsistent among studies [5, 6. This relationship is important to understand in guiding clinical practices aimed at optimizing operative time without compromising procedural efficacy [7]. Therefore, it is of interest to assess the effect of surgical duration on postoperative infection rates in a cohort of patients undergoing orthopedic procedures.

## Methodology:

This is an analytical study carried out in a tertiary care hospital over 12 months. It was to assess the relationship between surgical time and postoperative infection rates for orthopedic procedures. In total, 100 patients were selected who had undergone elective or emergency orthopedic surgeries. Patients with existing infections or undergoing revision surgeries were excluded to eliminate confounding factors. Data on surgical duration, type of procedure, the use of implants and infection rates post-surgery within 30 days were collected. Surgical duration was divided into three categories: less than 90 minutes, 90-180 minutes and more than 180 minutes. Postoperative infections were established based on clinical signs, laboratory tests and microbiological cultures. Descriptive statistics and inferential statistics were used to investigate the data gathered. Chi square tests were made to evaluate association between surgical length and infection prevalence. Logistic models, adjusted with potential confounding variables like comorbidities, age of patients, procedural complexity, in order to explain the relationship at hand. Obtain ethical permissions and ascertained informed consents from every participant.

## Results:

Table 1 (see PDF) outlines the demographic and clinical features of the study population. The mean age of participants was 46.5 years with slight gender predominance in males. Significant comorbidities, including diabetes and hypertension, were found in the patient population. Table 2 (see PDF) demonstrates the spread of infection rates by various surgical time lengths. The rates of infections were notably higher when surgeries lasted longer than 180 minutes. Table 3 (see PDF) emphasizes infection rates in accordance with the nature of orthopedic surgery. Replacements of the joints showed the highest rates of infections. Table 4 (see PDF) assesses several risk factors for postoperative infections. Longer operative times and diabetes were significant risk factors. Table 5 (see PDF) compared infection outcomes and the length of hospital stay associated with those infections. Patients with infections had significantly longer hospital stays. Table 6 (see PDF) summarized antibiotic use in infected and non-infected patients. Patients with infections required prolonged courses of antibiotics. The study population had a mean age of 46.5 years, with a predominance of men (58%) and significant comorbidities such as diabetes mellitus (22%) and hypertension (18%) (Table 1 - see PDF). Postoperative infection rates were closely correlated with surgical duration and increased from 5% for surgeries less than 90 minutes to 33% for surgeries longer than 180 minutes (Table 2 - see PDF). Among procedure types, joint replacement surgeries had the highest infection rate at 20%, followed by fracture fixation (8%) and soft tissue repair (5%) (Table 3 - see PDF). The main risk factors for infections were prolonged surgical duration (odds ratio: 3.5, p < 0.001) and diabetes mellitus (odds ratio: 2.8, p = 0.002) (Table 4 - see PDF). Infected patients were hospitalised for a much longer period of time, 12.7 days compared with the non-infected, 5.2 days, p < 0.001 (Table 5 - see PDF) and needed longer periods of antibiotic therapy, 14.8 days versus 5.1 days, p < 0.001 (Table 6 - see PDF). These data illustrate how the risk factors and surgical time influence postoperative infection following orthopedic surgery.

## Discussion:

The results of this study underscore the strong association between surgical duration and postoperative infection rates in orthopedic procedures [8]. Procedures longer than 180 minutes were significantly associated with much higher infection rates, consistent with prior research indicating risks associated with long operative times [9]. Long surgical times expose tissues to environmental pathogens for a longer period, compromise immune function through prolonged anesthesia and increase the risk of contamination [10]. The highest rate of infection after joint replacement operations is likely caused by the presence of implants due to their massive use, being more prone to infection [11]. More comorbidity, particularly diabetes mellitus, increased the likelihood of infection in these patients hence; adequate perioperative management should be meticulously conducted in those at substantial risk. Infection led to long hospital stays and prolonged antibiotic treatments, indicating considerable pressure on healthcare delivery and patient's recovery [12]. Such results point out that optimizing surgical efficiency and preventive measures through strict aseptic protocols, among others, would be able to minimize risks associated with infection. Future studies are encouraged to discuss operative time reduction methods that do not compromise surgical outcome and to evaluate other factors responsible for postoperative infections in diverse patient populations.

## Conclusion:

A strong association between long surgical duration and increased postoperative infection rates with orthopedic procedures is shown. Infections were at a significant risk when surgeries crossed the 180-minute mark, primarily among patients with comorbid conditions like diabetes mellitus. The optimization of surgical duration and stringent perioperative measures are critical to reducing infections, decreasing hospital stays and enhancing patient outcomes.
